# Identification of EMT-Related Gene Signatures to Predict the Prognosis of Patients With Endometrial Cancer

**DOI:** 10.3389/fgene.2020.582274

**Published:** 2020-12-02

**Authors:** Luya Cai, Chuan Hu, Shanshan Yu, Lixiao Liu, Jinduo Zhao, Ye Zhao, Fan Lin, Xuedan Du, Qiongjie Yu, Qinqin Xiao

**Affiliations:** ^1^Department of Obstetrics and Gynecology, The First Affiliated Hospital of Wenzhou Medical University, Wenzhou, China; ^2^Department of Orthopaedic Surgery, The Affiliated Hospital of Qingdao University, Qingdao, China; ^3^Department of Chemoradiation Oncology, The First Affiliated Hospital of Wenzhou Medical University, Wenzhou, China; ^4^Department of Dermatology, The First Affiliated Hospital of Wenzhou Medical University, Wenzhou, China; ^5^Department of Radiology, The First Affiliated Hospital of Wenzhou Medical University, Wenzhou, China

**Keywords:** endometrial cancer, EMT, prognosis, gene signature, nomogram

## Abstract

**Background:**

Endometrial cancer (EC) is one of the most common gynecological cancers. Epithelial–mesenchymal transition (EMT) is believed to be significantly associated with the malignant progression of tumors. However, there is no relevant study on the relationship between EMT-related gene (ERG) signatures and the prognosis of EC patients.

**Methods:**

We extracted the mRNA expression profiles of 543 tumor and 23 normal tissues from The Cancer Genome Atlas database. Then, we selected differentially expressed ERGs (DEERGs) among these mRNAs. Next, univariate and multivariate Cox regression analyses were performed to select the ERGs with predictive ability for the prognosis of EC patients. In addition, risk score models were constructed based on the selected genes to predict patients’ overall survival (OS), progression-free survival (PFS), and disease-free survival (DFS). Finally, nomograms were constructed to estimate the OS and PFS of EC patients, and pan-cancer analysis was performed to further analyze the functions of a certain gene.

**Results:**

Six OS-, ten PFS-, and five DFS-related ERGs were obtained. By constructing the prognostic risk score model, we found that the OS, PFS, and DFS of the high-risk group were notably poorer. Last, we found that AQP5 appeared in all three gene signatures, and through pan-cancer analysis, it was also found to play an important role in immunity in lower grade glioma (LGG), which may contribute to the poor prognosis of LGG patients.

**Conclusions:**

We constructed ERG signatures to predict the prognosis of EC patients using bioinformatics methods. Our findings provide a thorough understanding of the effect of EMT in patients with EC and provide new targets and ideas for individualized treatment, which has important clinical significance.

## Introduction

Endometrial cancer (EC) is one of the most common malignant tumors in women. According to global cancer statistics, in 2018, there were approximately 382,000 new cases of EC worldwide, with nearly 90,000 deaths (Bray et al., 2018). The incidence rate of EC has increased year by year, especially in developed countries, and EC has become the most prevalent cancer among gynecological malignancies (Miller et al., 2019; Siegel et al., 2020). Patients with early stage EC usually have a good prognosis; however, for patients with advanced and recurrent EC, treatment options are extremely limited, and side effects are more serious, with a 5-year survival rate of only 10–30% (Morice et al., 2016; Clarke et al., 2019). Therefore, it is necessary to identify efficient biomarkers and therapeutic targets to predict and improve the prognosis of patients with EC.

Epithelial–mesenchymal transition (EMT) is a biological process that transforms epithelial cells into stromal cells and is involved in embryogenesis, wound healing, and cancer progression (Nieto et al., 2016; Dongre and Weinberg, 2019; Yang J. et al., 2020). In tumors, EMT makes tumor cells more mobile and invasive and promotes cancer progression and metastasis, making them resistant to antitumor drugs (Marcucci et al., 2016). At present, some studies have found that molecular markers related to EMT were significantly related to the unfavorable clinical outcomes of patients with EC. For example, the lncRNA LINC01123 can promote the invasion and metastasis of EC by promoting the EMT pathway, which leads to disease progression and poor prognosis (Yang Y. et al., 2020). QKI, a circRNA regulator, was observed to have a mechanism that promotes EMT in EC, leading to poor clinical outcomes (Dou et al., 2020). Moreover, E-cadherin, an EMT-related protein, has decreased expression in EC, which represents an EMT-positive status that is significantly related to poor overall survival (OS) and progression-free survival (PFS) in patients with EC (Tanaka et al., 2013). However, single gene biomarkers cannot achieve a good prediction effect, and some studies have suggested that gene signatures may be a better choice for predicting patient outcomes.

**FIGURE 1:**
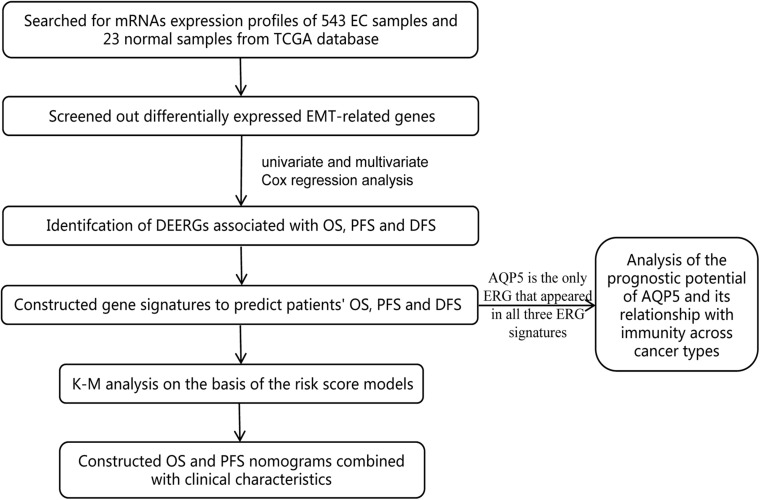
Flow chart of the bioinformatic analysis.

By mining public databases, a few scholars have studied the relationship between the EMT-related gene (ERG) signature and the prognosis of patients with cancer, such as glioma (Tao et al., 2020), gastric adenocarcinoma (Zhang et al., 2020), and head and neck squamous cell carcinoma (Kisoda et al., 2020). However, there is no bioinformatics research on this in EC. Therefore, in this study, we analyzed the relationship between the ERG signature and the prognosis of EC patients through The Cancer Genome Atlas (TCGA) database and constructed nomograms integrating clinical characteristics to estimate EC patients’ OS and PFS more conveniently. These findings help us better assess the prognosis of patients and provide new insights for the individualized treatment of patients with EC (Figure 1).

## Materials and Methods

### Patient Clinical Information and mRNA Expression Dataset

We downloaded the ERG list from the EMT gene database^1^ (Supplementary Table 1) and extracted the mRNA expression profiles of 543 EC samples and 23 normal samples from TCGA database^2^. The clinical information of the patients included age, grade, AJCC stage, histologic type, OS, PFS, and disease-free survival (DFS) (Supplementary Table 2).

### Identification and Analysis of Differentially Expressed ERGs (DEERGs)

We analyzed the expression levels of 1184 ERGs between EC and normal tissues with the limma package to screen out differentially expressed ERGs (DEERGs) and visualized them by volcano plots and heatmaps. False discovery rate <0.05 and | logFC| > 1 were defined as the significance threshold. Next, Gene Ontology (GO) and Kyoto Encyclopedia of Genes and Genomes (KEGG) pathway enrichment analyses were carried out on the basis of these DEERGs. Moreover, we used the STRING database to construct a protein–protein interaction (PPI) network of the selected genes to visualize the relationships between the DEERGs; 0.4 was defined as the minimum required interaction score, and genes with node degree >15 were selected as hub genes.

### Construction and Analysis of the Prognostic Signature

To identify the prognostic value of the DEERGs in EC patients, univariate Cox regression analysis was performed to confirm OS-, PFS-, and DFS-related DEERGs. Then, LASSO analysis was used to avoid collinearity and multivariable Cox analysis was used to construct the ERG-based prognostic signatures. The linear combination of the expression values of the selected genes weighted by their respective regression coefficient from the multivariate Cox regression analysis was used to establish the prognostic risk score model as follows: risk score = Σ (β*n* × expression of gene *n*).

Then, we divided the EC patients into high- and low-risk groups based on the median risk score. Kaplan–Meier (K-M) survival curves and the log-rank test were adopted to compare the prognostic differences between these two groups. In addition, we calculated receiver operating characteristic (ROC) curves to evaluate the discrimination of the prognostic model.

### Establishment of EMT-Clinical Nomograms

Nomograms can predict the survival rate of individual tumor patients based on the values of multiple variables. We combined the risk score with clinical characteristics and constructed nomograms to calculate the OS and PFS of patients with EC more conveniently, making the gene signature more practical. We performed univariate Cox regression analyses on the clinical data of the patients from the TCGA database to investigate which variables are significantly related to OS and PFS. Next, stepwise multivariate analysis was performed based on the variables we obtained. Finally, combining clinical characteristics and the ERG-based risk signature, we established a nomogram by using the rms package.

### Pan-Cancer Analysis of a Gene

According to the results of multivariable Cox analysis, we tried to determine whether there was a certain gene that affected the OS, PFS, and DFS of EC patients at the same time. If such a gene existed, we would analyze this gene through the UCSC database to observe its expression level in different tumor tissues. K-M plotter was used to analyze the relationship between this gene and the survival of various cancers, and log-rank *P* values and 95% CIs of the HR were calculated. In addition, we downloaded the scores of six immune-infiltrating cells in 33 cancers from the TIMER database and analyzed the correlation between the gene expression and the scores of these immune cells. We collected more than 40 common immune checkpoint genes and analyzed the relationship between gene expression and immune checkpoint gene expression.

## Results

### Preliminary Screening of ERGs

**FIGURE 2:**
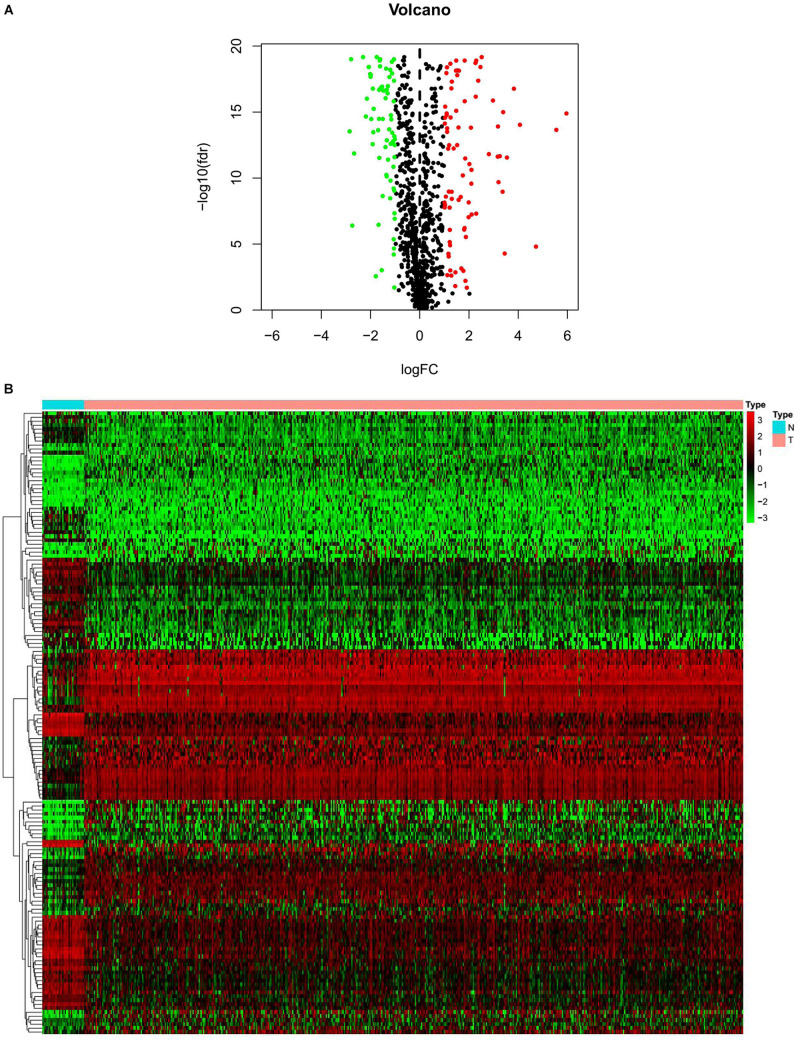
Differentially expressed EMT-related genes (ERGs) between endometrial cancer (EC) and normal tissues. **(A)** The volcano plot for the 1,184 ERGs from the TCGA-STAD cohort. **(B)** Heatmap for screened ERGs between 543 tumor samples and 23 normal samples.

**FIGURE 3:**
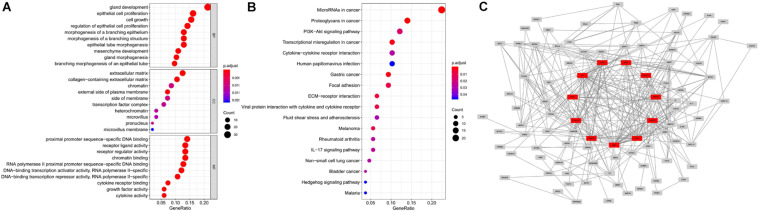
GO and KEGG pathway enrichment analysis and PPI network of DEERGs. **(A)** Functional annotation of DEERGs by GO enrichment analysis. **(B)** Functional annotation of DEERGs by KEGG pathway enrichment analysis. **(C)** PPI network analysis of 157 DEERGs.

We obtained 1,184 ERGs from the EMT gene database and the mRNA expression profiles from the TCGA database, including 543 tumor samples and 23 normal samples (Figures 2A,B). By comparing tumor and normal tissue samples, we screened out 157 DEERGs. To further analyze the biological functions and significant pathways of these DEERGs, we carried out GO analysis and KEGG pathway enrichment analysis on these genes. As a result, GO analysis revealed that the primary functions of the genes regarding biological processes (BPs) were gland development, epithelial cell proliferation, and cell growth. For the cellular component (CC) category, extracellular matrix and collagen-containing extracellular matrix were the main enriched GO terms. For the molecular function (MF) category, proximal promoter sequence-specific DNA binding, receptor ligand activity, and receptor regulator activity were the most enriched (Figure 3A). For KEGG pathway enrichment analysis, microRNAs in cancer, proteoglycans in cancer, and the PI3K-Akt signaling pathway were most often enriched by the DEERGs (Figure 3B). The PPI network of these DEERGs is shown in Figure 3C. The hub genes were EZH2, IL6, SPP1, CDKN2A, TWIST1, EGF, FGF2, RUNX2, WNT3A, TNFSF11, and BDNF, which are displayed in red.

### Identification of DEERGs Associated With Prognosis

**FIGURE 4:**
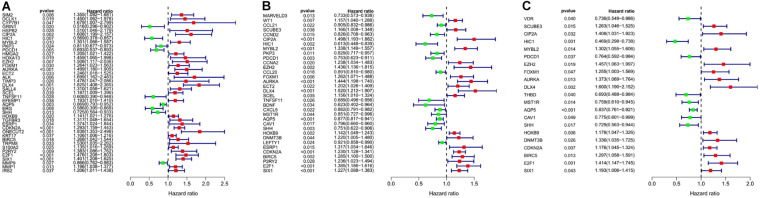
Forest plots of the prognosis-related ERGs by univariate Cox analysis. **(A)** OS-related ERGs. **(B)** PFS-related ERGs. **(C)** DFS-related ERGs. Green: protective factor, red: risk factor.

We analyzed the 157 DEERGs selected previously to screen out prognosis-related genes by univariate Cox regression analysis and found 41, 34, and 21 ERGs (*P* < 0.05) significantly associated with OS, PFS, and DFS, respectively (Figure 4). Next, we performed LASSO analysis (Supplementary Figure 1) and multivariable Cox analysis to further identify ERGs related to prognosis and obtained their respective coefficients. Finally, six ERGs (SIM2, PDCD1, AQP5, CDKN2A, ONECUT2, and SIX1), ten ERGs (MARVELD3, MYBL2, PKP3, PDCD1, BDNF, AQP5, CAV1, ESRP1, CDKN2A, and SIX1), and five ERGs (HIC1, MST1R, AQP5, HOXB9, and E2F1) that were associated with OS, PFS, and DFS, respectively, were obtained (Tables 1–3).

**TABLE 1:** Information on six prognostic ERGs significantly related to OS in patients with EC, which were used to constructed the final prediction model.

mRNA	B (Cox)	HR	*P*
SIM2	0.2542	1.2894	0.0208
PDCD1	−0.2817	0.7545	0.0330
AQP5	−0.1325	0.8759	0.0086
CDKN2A	0.1565	1.1695	0.0078
ONECUT2	0.3562	1.4279	0.0365
SIX1	0.2257	1.2532	0.0048

**TABLE 2:** Information on ten prognostic ERGs significantly related to PFS in patients with EC, which were used to constructed the final prediction model.

mRNA	B (Cox)	HR	*P*
MARVELD3	−0.2824	0.7540	0.0764
MYBL2	0.1396	1.1498	0.1024
PKP3	−0.2229	0.8002	0.0054
PDCD1	−0.1611	0.8512	0.1016
BDNF	−0.4211	0.6563	0.0568
AQP5	−0.1074	0.8982	0.0098
CAV1	−0.1604	0.8518	0.1018
ESRP1	0.3390	1.4035	0.0014
CDKN2A	0.0869	1.0908	0.0715
SIX1	0.1289	1.1375	0.0527

**TABLE 3:** Information on five prognostic ERGs significantly related to DFS in patients with EC, which were used to constructed the final prediction model.

mRNA	B (Cox)	HR	*P*
HIC1	−0.5554	0.5738	0.0181
MST1R	−0.208	0.8122	0.0601
AQP5	−0.1122	0.8939	0.0295
HOXB9	0.1114	1.1179	0.0538
E2F1	0.2403	1.2717	0.0332

### Constructing Three ERG Signatures to Predict Patient Prognosis

**FIGURE 5:**
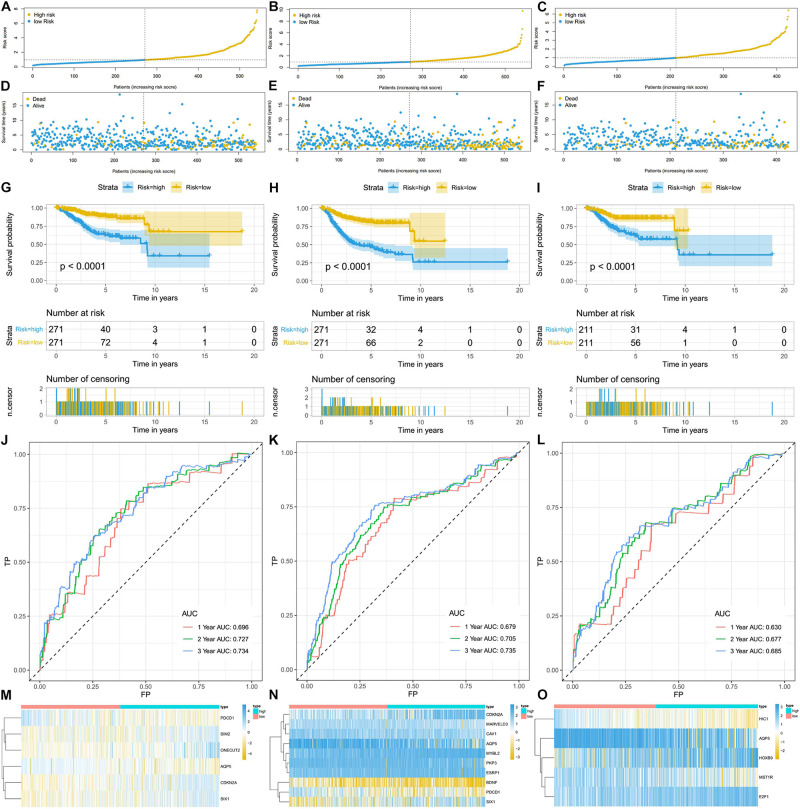
The ERG signatures associated with risk score predicts OS, PFS, and DFS in patients with EC. **(A–C)** The risk score distribution of EC patients. **(D–F)** Survival status and survival times of EC patients. **(G–I)** K-M curve to test the predictive effect of the gene signature. **(J–L)** ROC curve analysis of risk score with survival time at 1, 2, and 3 years to evaluate the sensitivity and specificity of the gene signature. **(M–O)** A heatmap of the expression profile of ERGs, which constructed gene signatures. Red: low-risk group, blue: high-risk group. Color from yellow to blue indicates gene expression from low to high.

Using the linear combination of the expression value of the selected genes and their respective regression coefficient from multiple Cox regression analysis, we established three risk scoring models to predict EC patients’ prognosis (OS, PFS, and DFS). Based on the prognosis risk score, the EC patients were divided into high- and low-risk groups by using the median risk score as the cutoff value (Figures 5A–C), and the respective survival status of the EC patients was obtained (Figures 5D–F). The K-M analysis showed that compared with those of the high-risk group, the OS, PFS, and DFS of the low-risk group were markedly better (*P* < 0.0001; Figures 5G–I). The AUCs for 1-, 3-, and 5-year OS, PFS, and DFS were all in the range of 0.630–0.735 (Figures 5J–L), suggesting that these three ERG signatures have excellent diagnostic significance for prognosis prediction. In addition, we generated heatmaps to exhibit the expression profiles of these three groups of ERGs, visualizing the expression of the ERGs in the high- and low-risk groups (Figures 5M–O).

### Developed Nomograms Integrating Clinical Characteristics Showed That Several Clinical Characteristics Were Significantly Related to the Risk Score

**FIGURE 6:**
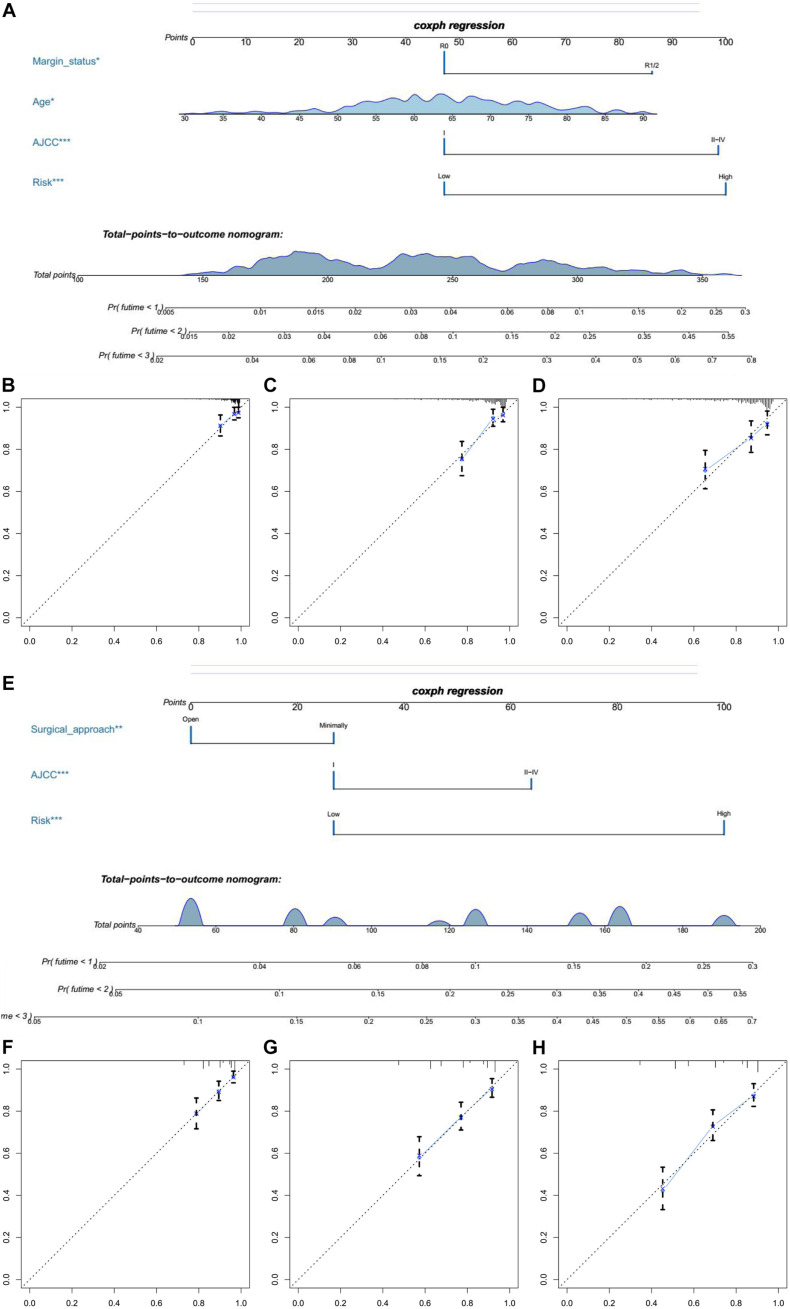
The establishment of two nomograms which can predict the prognosis probability of EC patients. **(A)** OS nomogram was constructed combined with risk score and three clinical characteristic (margin status, age, and AJCC stage). **(B–D)** The calibration plot of OS nomogram for 1-, 2-, and 3-year survival. **(E)** PFS nomogram was constructed combined with risk score and two clinical characteristic (surgical approach and AJCC stage). **(F–H)** The calibration plot of PFS nomogram for 1-, 2-, and 3-year PFS.

In univariate Cox regression analyses, we found that risk score, age, AJCC stage, grade, histologic type, and margin status were significantly associated with the OS and PFS of EC patients, and race, hypertension, and surgical approach can also significantly influence patients’ PFS (Table 4). Then, we further performed multivariate Cox regression analyses and the results showed that risk score (HR 2.41; 95% CI 1.32 to 4.40; *P* = 0.004), age (HR 1.03; 95% CI 1.00 to 1.05; *P* = 0.032), AJCC stage (HR 2.57; 95% CI 1.46 to 4.54; *P* = 0.001), and margin status (HR 1.94; 95% CI 1.04 to 3.64; *P* = 0.039) were independently related to OS (Table 5). Based on these independent survival indicators, we constructed a nomogram prediction model to predict EC patients’ OS (Figure 6A). In addition, another nomogram was constructed to predict the PFS of patients with EC (Figure 6E), in which the risk score (HR 4.12; 95% CI 2.38 to 7.12; *P* < 0.001), AJCC stage (HR 1.76; 95% CI 1.13 to 2.73; *P* = 0.012), and surgical approach (HR 0.62; 95% CI 0.40 to 0.96; *P* = 0.033) were integrated as independent risk factors (Table 5). The calibration plot showed that in these two nomograms, the predicted values of OS or PFS at 1, 2, and 3 years for EC patients have a good correlation with the actual values (Figures 6B–D, F–H).

**TABLE 4:** Univariate Cox regression analyses for identifying clinical characteristics related to EC patients’ OS and PFS.

Clinical feature	Univariate analysis (OS)			Univariate analysis (PFS)		
	HR	95%CI of HR	*P* value	HR	95%CI of HR	*P* value
Risk score	3.664	2.280–5.888	<0.001	3.707	2.570–5.348	<0.001
Age, y	1.034	1.014–1.054	<0.001	1.021	1.006–1.036	0.006
**Race**						
Black	1 (reference)			1 (reference)		
Other	0.540	0.201–1.450	0.222	0.350	0.136–0.902	0.030
White	0.876	0.525–1.461	0.612	0.913	0.612–1.363	0.658
**AJCC stage**						
I	1 (reference)			1 (reference)		
II-IV	3.667	2.377–5.657	<0.001	2.231	1.616–3.078	<0.001
**Grade**						
Low	1 (reference)			1 (reference)		
High	3.413	1.988–5.859	<0.001	1.877	1.306–2.696	<0.001
**Histologic_type**						
EEA	1 (reference)			1 (reference)		
MSE	2.880	1.230–6.744	0.015	1.977	0.959–4.078	0.065
SEA	2.895	1.878–4.461	<0.001	2.049	1.450–2.894	<0.001
**Hypertension**						
NO	1 (reference)			1 (reference)		
YES	0.282	0.047–1.699	0.167	0.203	0.053–0.770	0.019
**Radiotherapy**						
NO	1 (reference)			1 (reference)		
YES	0.448	0.071–2.832	0.394	0.685	0.170–2.761	0.594
**Margin_status**						
R0	1 (reference)			1 (reference)		
R1/2	3.261	1.852–5.744	<0.001	2.192	1.319–3.643	0.002
**Surgical_approach**						
Minimally Invasive	1 (reference)			1 (reference)		
Open	0.753	0.489–1.160	0.198	0.670	0.480–0.936	0.019

**TABLE 5:** Multivariate Cox regression analyses for identifying independent clinical characteristics related to EC patients’ OS and PFS.

Clinical feature	Multivariate analysis (OS)			Multivariate analysis (PFS)		
	HR	95%CI of HR	*P* value	HR	95%CI of HR	*P* value
Risk score	2.413	1.324–4.398	0.004	4.120	2.383–7.122	<0.001
Age, y	1.030	1.002–1.052	0.032	0.999	0.978–1.020	0.910
**Race**						
Black	–	–	–	1 (reference)		
Other	–	–	–	0.511	0.164–1.590	0.246
White	–	–	–	1.400	0.822–2.559	0.246
**AJCC stage**						
I	1 (reference)			1 (reference)		
II-IV	2.572	1.459–4.536	0.001	1.756	1.130–2.729	0.012
**Grade**						
Low	1 (reference)			1 (reference)		
High	1.464	0.739–2.900	0.274	0.638	0.367–1.110	0.112
**Histologic_type**						
EEA	1 (reference)			1 (reference)		
MSE	1.237	0.426–3.592	0.696	1.318	0.497–3.495	0.579
SEA	0.875	0.485–1.581	0.659	1.193	0.700–2.035	0.516
**Margin_status**						
R0	1 (reference)			1 (reference)		
R1/2	1.941	1.035–3.641	0.039	1.691	0.946–3.020	0.076
**Surgical_approach**						
Minimally Invasive	–	–	–	1 (reference)		
Open	–	–	–	0.622	0.402–0.963	0.033

**FIGURE 7:**
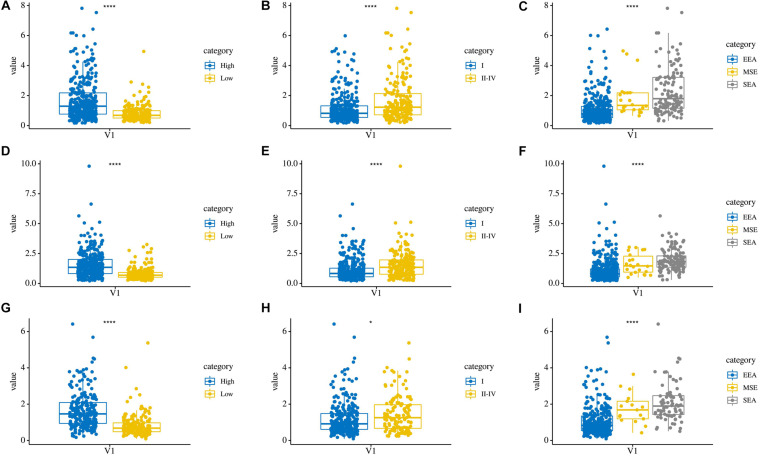
The relationship between risk score and clinical characteristics. **(A–C)** Distribution of OS risk scores in different grades, AJCC stages, and histological types. **(D–F)** Distribution of PFS risk scores in different grades, AJCC stages, and histological types. **(G–I)** Distribution of DFS risk scores in different grades, AJCC stages, and histological types.

In addition, we compared the relationship between the risk score and three clinicopathological features: grade, AJCC stage, and histological type, which were significantly related to both OS, PFS, and DFS. Patients in the high grade, AJCC stage II–IV, and serous endometrial adenocarcinoma groups had higher risk scores (*P* < 0.0001) in these three risk score models (Figures 7A–I). These results showed that the risk score is closely related to clinical characteristics.

### Prognostic Potential of AQP5 and Its Relationship With Immunity Across Cancer Types

**FIGURE 8:**
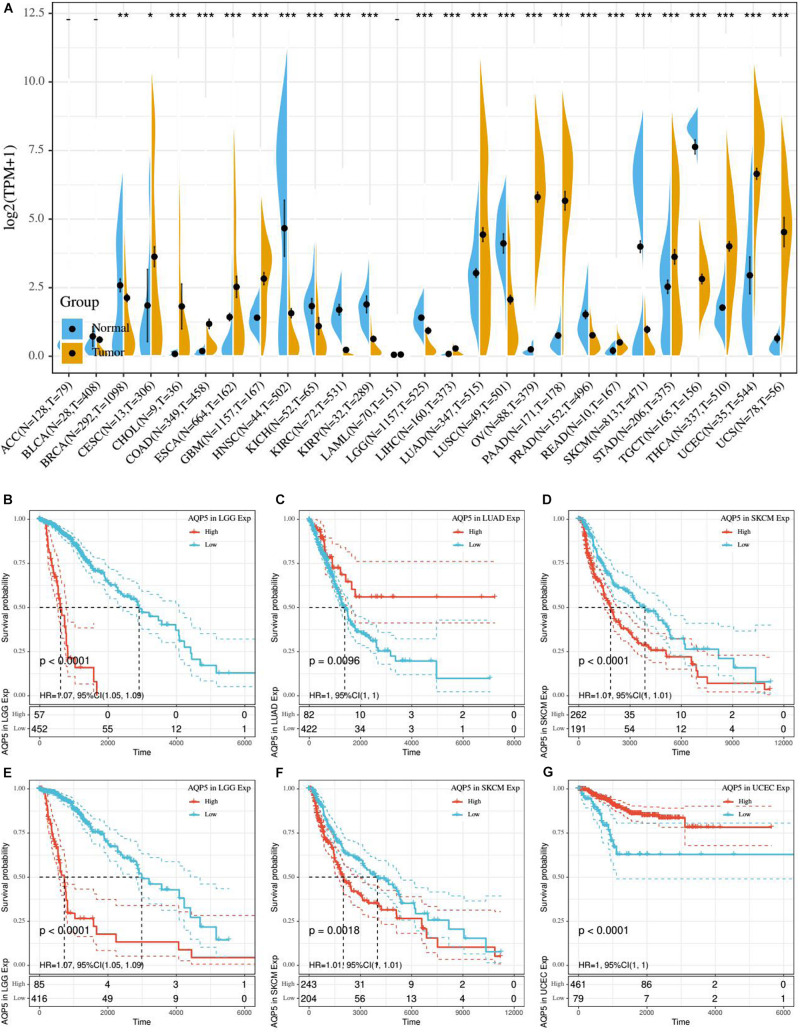
Gene expression correlation analysis and prognostic potential of AQP5. **(A)** Expression difference of AQP5 in 27 cancer types through UCSC database. **(B–D)** Analysis of the relationship between AQP5 expression and OS of patients in LGG, LUAD, and SKCM by K-M survival curves. **(E–G)** Analysis of the relationship between AQP5 expression and DSS of patients in LGG, SKCM, and UCEC by K-M survival curves. OS, overall survival; DSS, disease-specific survival. “–,” not significant, *P < 0.05, **P < 0.01, and ***P < 0.001. *P* < 0.05 was considered to be statistically significant.

Based on the aforementioned results, we found that AQP5 is the only ERG that appeared in all three ERG signatures. Therefore, we further analyzed AQP5 to determine whether it has a certain effect on prognosis in other cancer types. First, we performed gene expression correlation analysis using the samples from the UCSC database and found that in most cancer types, there were differences in the expression level, which could be higher or lower in tumor tissues, except for in ACC, BLCA, and LAML (Figure 8A). Subsequently, we compared the relationship between the expression level of AQP5 and prognosis in 33 kinds of cancers from the TCGA database. The results showed that AQP5 expression in lower grade glioma (LGG), LUAD, and SKCM was significantly different for the OS of patients (Figures 8B–D), and in LGG, SKCM, and UCEC, it was significantly different for the disease-specific survival (DSS) of patients (Figures 8E–G). Moreover, in LGG and SKCM, the high expression of AQP5 was associated with worse OS and DSS (Figures 8B,D–F), whereas in LUAD, the high expression of AQP5 represented better OS with *P* = 0.0096 (Figure 8C) and in UCEC, the high expression of AQP5 represented better DSS with *P* < 0.001 (Figure 8G).

**FIGURE 9:**
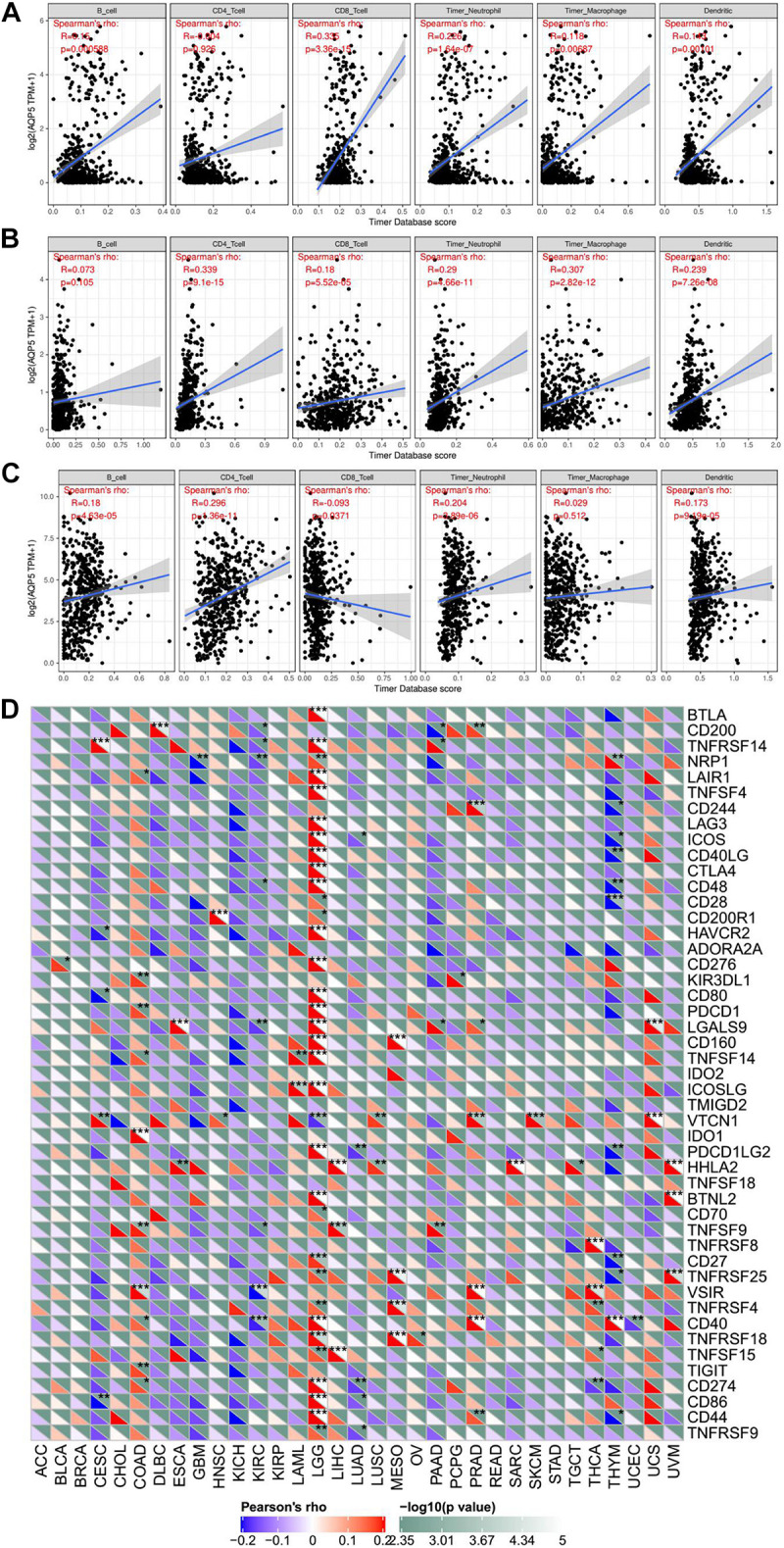
The relationship between AQP5 and immunity across cancer types. **(A–C)** Analysis of the relationship between AQP5 expression and the level of immune infiltration in LGG, PRAD, and THCA by TIMER database. **(D)** Relationship between AQP5 expression and the immune checkpoint gene expression.

In addition, we analyzed the relationship between the expression of AQP5 and the immune response in 33 cancer types. As a result, we found that the expression of AQP5 in LGG, PRAD, and THCA was significantly correlated with the level of immune infiltration (Figures 9A–C). Interestingly, in LGG, the higher expression level of AQP5 was related to poorer prognosis and higher immune infiltration levels of B cells (*R* = 0.15, *P* = 0.00059), CD8^+^ cells (*R* = 0.305, *P* = 3.36e-15), DC cells (*R* = 0.143, *P* = 0.00101), macrophages (*R* = 0.116, *P* = 0.00687), and neutrophils (*R* = 0.226, *P* = 1.64e-07) (Figure 9A). Moreover, in the analysis of the relationship between the expression of AQP5 and immune checkpoint gene expression, we found that in LGG, the AQP5 expression level had a significant positive correlation with the expression of most immune checkpoint genes (Figure 9D). These findings strongly suggest that AQP5 plays a prominent role in immunity in LGG, which may contribute to the poor prognosis of patients.

## Discussion

In recent decades, increasing evidence has shown that EMT is closely related to the progression, metastasis, and drug resistance of cancers (Nieto et al., 2016). In tumors, EMT is activated, and the most important sign of this is the downregulation of E-cadherin. E-cadherin is a protein that spans the cell membrane and closely binds adjacent cells; it is an important molecule for maintaining the characteristics of epithelial cells. Its loss or downregulation promotes the distant dissemination of cancer cells (Thiery et al., 2009; Lamouille et al., 2014). In addition, related transcription factors, such as SNAIL, TWIST, and zinc finger E-box binding (ZEB), also play significant roles in the biological process of EMT, promoting cell invasion, migration, proliferation, and angiogenesis (Dongre and Weinberg, 2019). At present, many studies have indicated that EMT status is closely associated with the survival of cancer patients, and some ERG signatures have been constructed to predict the survival of patients with cancer. For example, in non-small cell lung cancer, E-cadherin was found to interact with epidermal growth factor receptor, playing a pivotal role in prognosis and progression (Witta et al., 2006). An EMT-related seven-gene signature was used to predict the survival of patients with glioma (Tao et al., 2020). Furthermore, Tan et al. (2014), found that EMT status was related to OS and DFS in patients with ovarian cancer, and EMT scoring was performed. However, studies on the relationship between ERGs and the prognosis of patients with EC are still very limited. Because a single gene biomarker cannot provide a strong prediction effect, a more accurate and reliable gene signature was used to predict the clinical outcomes of patients. In this study, we used the ERG signature for the first time to predict the survival of patients with EC and obtained a good prediction effect.

First, we screened out the DEERGs between 543 EC samples and 23 normal samples from the TCGA database and selected OS-related, PFS-related, and DFS-related DEERGs with predictive ability for the prognosis of patients with EC through univariate and multivariate Cox regression analyses (*P* < 0.05). Subsequently, by constructing the prognostic risk score models, we found that there were significant differences in OS, PFS, and DFS between the high- and low-risk groups, and the OS, PFS, and DFS of the high-risk group were evidently worse with *P* < 0.0001. In addition, we constructed two nomograms integrating clinical characteristics that provide a more convenient way to estimate the OS and PFS of patients with EC. Among them, AJCC stage was an independent risk factor used to construct both of the nomograms. Of course, the independent prognostic indicators we obtained are to fully consider the existing data, and the possible influencing factors have been investigated one by one. However, there is no denying that many may be confused by our conclusion that clinical data may not be included in the research, but we believe that with the increasing improvement of database data, as well as our research that is gradually thorough, the future will be more objective to front the dialectical point of view to look at the impact of these clinical indices for prognosis.

Moreover, according to our analysis, we found that AQP5 is the only ERG related to EC patients’ OS, PFS, and DFS. AQP5 is a kind of transmembrane water channel protein that plays pivotal roles in regulating the water balance in cells and in maintaining cell function and has been found in a variety of tumors. Many studies have proven that AQP5 is closely associated with the migration and proliferation of cancer cells, and it can become a prognostic marker and potential drug target. For example, in colorectal cancer, the overexpression of AQP5 can promote the invasion and migration of cancer cells by activating EMT (Chen et al., 2017). There are also many related studies in ovarian cancer, and the expression of AQP5 in ovarian cancer is significantly increased, which is consistent with our previous gene expression correlation analysis using samples from the UCSC database, and is associated with the formation of ascites (Yang et al., 2006; Tiwari et al., 2014). In addition, the downregulation of AQP5 expression can inhibit ovarian cancer development (Yan et al., 2014). Furthermore, AQP5 also plays a key role in cervical cancer; its high expression is positively correlated with the expression of Ki-67, and both are significantly correlated with lymph node metastasis and poor prognosis (Zhang et al., 2012). On the basis of our pan-cancer analysis, in LGG and SKCM, the high expression of AQP5 was related to worse OS and DSS, which is consistent with the hypothesis that AQP5 may promote the proliferation and metastasis of tumor cells. However, in LUAD and UCEC, the high expression of AQP5 was associated with a better prognosis. What is more, in our study, AQP5 is found to be a protective factor for EC patients. Therefore, we suggested that AQP5 may play different roles in different cancer types with different mechanisms, and that in certain cancers may promote the proliferation of tumor cells and in certain cancers may inhibit the growth of tumor cells, but the specific mechanism is not clear. In our study, although AQP5 plays a protective role in patients with EC, there are no relevant studies that have reported its specific molecular mechanism in EC, and further in-depth studies are needed.

In addition to AQP5, we also found that only PDCD1, SIX1, and ONECUT2 are ERGs that were repeatedly found in the gene signatures related to OS and PFS in EC patients. PDCD1 is a gene that encodes programmed cell death protein 1 (PD-1). PD-1 is an important immunosuppressive molecule, and its ligands are PD-L1 and PD-L2. The high expression of PD-1 in EC is positively correlated with high levels of microsatellite instability (MSI) and better OS (Kim et al., 2018; Sungu et al., 2019), which are consistent with our multivariable Cox analysis that PDCD1 is a protective factor with hazard ratio (HR) <1. Oncofetal protein sine oculis-related homeobox 1 (SIX1) is a transcription factor that plays a key role in the proliferation and development of tumor cells and is a development EMT regulator (Christensen et al., 2008; Micalizzi et al., 2010; Wu et al., 2015). In EC, SIX1 also plays an important role, and as a disease biomarker (Suen et al., 2016), its overexpression can promote the growth of tumor cells through ERK- and AKT-mediated pathways (Xin et al., 2016). One cut homeobox 2 (ONECUT2) is a transcription factor related to tumor cell proliferation, which is closely associated with the EMT process of cancer cells (Sun et al., 2014; Wang et al., 2020). Some studies have found that its expression is significantly related to the progression of ovarian cancer (Lu et al., 2018), prostate cancer (Joglekar et al., 2020), lung adenocarcinoma (Ma et al., 2019), etc. However, there are few studies that have studied the role of this gene in EC.

It is undeniable that this study does have some limitations. On the one hand, the prediction models constructed by the genes we selected need to be independently validated before they can be used to evaluate the prognosis of patients with EC. Unfortunately, the GEO database lacks an EC dataset with OS, PFS, or DFS, so the stability of the prognostic models has not been verified. On the other hand, there is a limitation that we did not integrate multi-omics data which would improve identification accuracy and prediction performance of prognostic models. Last but not least, in this paper, Cox regression is used to screen variables and establish a prediction model, which is a statistical method widely used in survival analysis. However, in recent years, with the development of science and technology, many better algorithms are gradually developed (Wu and Ma, 2015; Wu et al., 2018; Ren et al., 2019). We also hope that these methods applied to the prognosis of tumor-related research in the future.

In summary, we first identified the relationship between the ERG signature and the prognosis of patients with EC using bioinformatics methods and found that patients in the high-risk group had significantly lower OS, PFS, and DFS rates than those in the low-risk group. Furthermore, we found that the ERG AQP5, which is related to OS, PFS, and DFS in patients with EC, has a close relationship with the prognosis of LGG patients.

## Conclusion

In brief, we constructed ERG signatures to predict the prognosis of patients with EC and built nomograms to estimate the prognosis of EC patients more accurately. In addition, AQP5 was the ERG that affected EC patients’ OS, PFS, and DFS, and we further explored its role in other cancer types through pan-cancer analysis. These findings provide new insights into the role of EMT in EC, guiding individualized treatment for patients with EC.

## Data Availability Statement

The original contributions presented in the study are included in the article/Supplementary Material, further inquiries can be directed to the corresponding author/s.

## Author Contributions

QX conceived and designed the study with LC and CH. LC and SY drafted the manuscript and analyzed the data with LL and CH. JZ and YZ handled the picture and manuscript format. FL, XD, and QY reviewed the data. All authors have read and approved the final published manuscript.

## Conflict of Interest

The authors declare that the research was conducted in the absence of any commercial or financial relationships that could be construed as a potential conflict of interest.

## Footnotes

1 http://dbemt.bioinfo-minzhao.org/download.cgi

2 https://www.cancer.gov/

## Supplementary Material

Supplementary Figure 1 LASSO Cox regression based on OS, PFS, and DFS. **(A–C)** LASSO coefficient profiles of the candidate OS-, PFS-, and DFS-related ERGs. **(D–F)** Dotted vertical lines were drawn at the optimal values by using the minimum criteria.

Supplementary Table 1 The ERG list from the epithelial–mesenchymal transition gene database.

Supplementary Table 2 The clinical information of the patients.

## References

[B1] Bray F. Ferlay J. Soerjomataram I. Siegel R. L. Torre L. A. Jemal A. (2018). Global cancer statistics 2018: GLOBOCAN estimates of incidence and mortality worldwide for 36 cancers in 185 countries. *CA Cancer J. Clin.* 68 394–424. 10.3322/caac.21492 30207593

[B2] Chen C. Ma T. Zhang C. Zhang H. Bai L. Kong L. (2017). Down-regulation of aquaporin 5-mediated epithelial-mesenchymal transition and anti-metastatic effect by natural product Cairicoside E in colorectal cancer. *Mol. Carcinog.* 56 2692–2705. 10.1002/mc.22712 28833571

[B3] Christensen K. L. Patrick A. N. McCoy E. L. Ford H. L. (2008). Chapter 5 the six family of homeobox genes in development and Cancer. *Adv. Cancer Res.* 101 93–126. 10.1016/s0065-230x(08)00405-3 19055944

[B4] Clarke M. A. Devesa S. S. Harvey S. V. Wentzensen N. (2019). Hysterectomy-corrected uterine corpus cancer incidence trends and differences in relative survival reveal racial disparities and rising rates of nonendometrioid Cancers. *J. Clin. Oncol.* 37 1895–1908. 10.1200/JCO.19 31116674 PMC6675596

[B5] Dongre A. Weinberg R. A. (2019). New insights into the mechanisms of epithelial-mesenchymal transition and implications for cancer. *Nat. Rev. Mol. Cell Biol.* 20 69–84. 10.1038/s41580-018-0080-4 30459476

[B6] Dou Y. Kawaler E. A. Cui Zhou D. Gritsenko M. A. Huang C. Blumenberg L. (2020). Proteogenomic characterization of endometrial carcinoma. *Cell* 180 729.e26–748.e26. 10.1016/j.cell.2020.01.026 32059776 PMC7233456

[B7] Joglekar T. Lin J. Shibata M. (2020). ONECUT2 is a novel target for treatment of castration-resistant prostate cancer. *Expert Opin. Ther. Targets* 24 89–90. 10.1080/14728222.2020.1723080 31983247 PMC7523780

[B8] Kim J. Kim S. Lee H. S. Yang W. Cho H. Chay D. B. (2018). Prognostic implication of programmed cell death 1 protein and its ligand expressions in endometrial cancer. *Gynecol. Oncol.* 149 381–387. 10.1016/j.ygyno.2018.02.013 29572029

[B9] Kisoda S. Shao W. Fujiwara N. Mouri Y. T. Tsunematsu Y. Jin S. (2020). Prognostic value of partial EMT-related genes in head and neck squamous cell carcinoma by a bioinformatic analysis. *Ora.l Dis*. 26 1149–1156. 10.1111/odi.13351 32277532

[B10] Lamouille S. Xu J. Derynck R. (2014). Molecular mechanisms of epithelial-mesenchymal transition. *Nat. Rev. Mol. Cell Biol.* 15 178–196. 10.1038/nrm3758 24556840 PMC4240281

[B11] Lu T. Wu B. Yu Y. Zhu W. Zhang S. Zhang Y. (2018). Blockade of ONECUT2 expression in ovarian cancer inhibited tumor cell proliferation, migration, invasion and angiogenesis. *Cancer Sci.* 109 2221–2234. 10.1111/cas.13633 29737581 PMC6029829

[B12] Ma Q. Wu K. Li H. Li H. Zhu Y. Hu G. (2019). ONECUT2 overexpression promotes RAS-driven lung adenocarcinoma progression. *Sci. Rep.* 9:20021. 10.1038/s41598-019-56277-2 31882655 PMC6934839

[B13] Marcucci F. Stassi G. De Maria R. (2016). Epithelial-mesenchymal transition: a new target in anticancer drug discovery. *Nat. Rev. Drug Discov.* 15 311–325. 10.1038/nrd.2015.13 26822829

[B14] Micalizzi D. S. Farabaugh S. M. Ford H. L. (2010). Epithelial-mesenchymal transition in cancer: parallels between normal development and tumor progression. *J. Mammary Gland Biol. Neoplasia* 15 117–134. 10.1007/s10911-010-9178-9 20490631 PMC2886089

[B15] Miller K. D. Nogueira L. Mariotto A. B. Rowland J. H. Yabroff K. R. Alfano C. M. (2019). Cancer treatment and survivorship statistics, 2019. *CA Cancer J. Clin.* 69 363–385. 10.3322/caac.21565 31184787

[B16] Morice P. Leary A. Creutzberg C. Abu-Rustum N. Darai E. (2016). Endometrial cancer. *Lancet* 387 1094–1108. 10.1016/s0140-6736(15)00130-0 26354523

[B17] Nieto M. A. Huang R. Y. Jackson R. A. Thiery J. P. (2016). Emt: 2016. *Cell* 166 21–45. 10.1016/j.cell.2016.06.028 27368099

[B18] Ren J. Du Y. Li S. Ma S. Jiang Y. Wu C. (2019). Robust network-based regularization and variable selection for high-dimensional genomic data in cancer prognosis. *Genet. Epidemiol.* 43 276–291. 10.1002/gepi.22194 30746793 PMC6446588

[B19] Siegel R. L. Miller K. D. Jemal A. (2020). Cancer statistics, 2020. *CA Cancer J. Clin.* 70 7–30. 10.3322/caac.21590 31912902

[B20] Suen A. A. Jefferson W. N. Wood C. E. Padilla-Banks E. Bae-Jump V. L. Williams C. J. (2016). SIX1 oncoprotein as a biomarker in a model of hormonal carcinogenesis and in human endometrial Cancer. *Mol. Cancer Res.* 14 849–858. 10.1158/1541-7786.MCR-16-0084 27259717 PMC5025359

[B21] Sun Y. Shen S. Liu X. Tang H. Wang Z. Yu Z. (2014). MiR-429 inhibits cells growth and invasion and regulates EMT-related marker genes by targeting Onecut2 in colorectal carcinoma. *Mol. Cell. Biochem.* 390 19–30. 10.1007/s11010-013-1950-x 24402783 PMC3972435

[B22] Sungu N. Yildirim M. Desdicioglu R. Basaran Aydogdu O. Kilicarslan A. Tatli Dogan H. (2019). Expression of immunomodulatory molecules PD-1, PD-L1, and PD-L2, and their relationship with clinicopathologic characteristics in endometrial Cancer. *Int. J. Gynecol. Pathol.* 38 404–413. 10.1097/PGP.0000000000000543 30134343

[B23] Tan T. Z. Miow Q. H. Miki Y. Noda T. Mori S. Huang R. Y. (2014). Epithelial-mesenchymal transition spectrum quantification and its efficacy in deciphering survival and drug responses of cancer patients. *EMBO Mol. Med.* 6 1279–1293. 10.15252/emmm.201404208 25214461 PMC4287932

[B24] Tanaka Y. Terai Y. Kawaguchi H. Fujiwara S. Yoo S. Tsunetoh S. (2013). Prognostic impact of EMT (epithelial-mesenchymal-transition)-related protein expression in endometrial cancer. *Cancer Biol. Ther.* 14 13–19. 10.4161/cbt.22625 23114646 PMC3566047

[B25] Tao C. Huang K. Shi J. Hu H. Li K. Zhu X. (2020). Genomics and prognosis analysis of epithelial-mesenchymal transition in glioma. *Front. Oncol.* 10:183. 10.3389/fonc.2020.00183 32154177 PMC7047417

[B26] Thiery J. P. Acloque H. Huang R. Y. Nieto M. A. (2009). Epithelial-mesenchymal transitions in development and disease. *Cell* 139 871–890. 10.1016/j.cell.2009.11.007 19945376

[B27] Tiwari A. Hadley J. A. Ramachandran R. (2014). Aquaporin 5 expression is altered in ovarian tumors and ascites-derived ovarian tumor cells in the chicken model of ovarian tumor. *J. Ovarian Res.* 7:99. 10.1186/s13048-014-0099-x PMC4213468 25344048

[B28] Wang G. H. Zhou Y. M. Yu Z. Deng J. P. Liu S. F. Wei C. Z. (2020). Up-regulated ONECUT2 and down-regulated SST promote gastric cell migration, invasion, epithelial-mesenchymal transition and tumor growth in gastric cancer. *Eur. Rev. Med. Pharmacol. Sci.* 18 9378–9390. 10.26355/eurrev_202009_23021 33015779

[B29] Witta S. E. Gemmill R. M. Hirsch F. R. Coldren C. D. Hedman K. Ravdel L. (2006). Restoring E-cadherin expression increases sensitivity to epidermal growth factor receptor inhibitors in lung cancer cell lines. *Cancer Res.* 66 944–950. 10.1158/0008-5472.CAN-05-1988 16424029

[B30] Wu C. Ma S. (2015). A selective review of robust variable selection with applications in bioinformatics. *Brief. Bioinform.* 16 873–883. 10.1093/bib/bbu046 25479793 PMC4570200

[B31] Wu C. Zhang Q. Jiang Y. Ma S. (2018). Robust network-based analysis of the associations between (epi)genetic measurements. *J. Mult. Ana.* 168 119–130. 10.1016/j.jmva.2018.06.009 30983643 PMC6456078

[B32] Wu W. Ren Z. Li P. Yu D. Chen J. Huang R. (2015). Six1: a critical transcription factor in tumorigenesis. *Int. J. Cancer* 136 1245–1253. 10.1002/ijc.28755 24488862

[B33] Xin X. Li Y. Yang X. (2016). SIX1 is overexpressed in endometrial carcinoma and promotes the malignant behavior of cancer cells through ERK and AKT signaling. *Oncol. Lett.* 12 3435–3440. 10.3892/ol.2016.5098 27900017 PMC5103964

[B34] Yan C. X. Zhu Y. Zhang X. Chen X. Zheng W. Yang J. (2014). Down-regulated aquaporin 5 inhibits proliferation and migration of human epithelial ovarian cancer 3AO cells. *J. Ovarian Res.* 7:78. 10.1186/s13048-014-0078-2 PMC4164796 25298246

[B35] Yang J. Antin P. Berx G. Blanpain C. Brabletz T. Bronner M. (2020). Guidelines and definitions for research on epithelial-mesenchymal transition. *Nat. Rev. Mol. Cell Biol.* 21 341–352. 10.1038/s41580-020-0237-9 32300252 PMC7250738

[B36] Yang Y. Wu J. Zhou H. Liu W. Wang J. Zhang Q. (2020). STAT1-induced upregulation of lncRNA LINC01123 predicts poor prognosis and promotes the progression of endometrial cancer through miR-516b/KIF4A. *Cell Cycle* 19 1502–1516. 10.1080/15384101.2020.1757936 32401659 PMC7469438

[B37] Yang J. H. Shi Y. F. Cheng Q. Deng L. (2006). Expression and localization of aquaporin-5 in the epithelial ovarian tumors. *Gynecol. Oncol.* 100 294–299. 10.1016/j.ygyno.2005.08.054 16242760

[B38] Zhang D. Zhou S. Liu B. (2020). Identification and validation of an individualized EMT-related prognostic risk score formula in gastric adenocarcinoma patients. *Biomed Res. Int.* 2020:7082408. 10.1155/2020/7082408 32309437 PMC7142392

[B39] Zhang T. Zhao C. Chen D. Zhou Z. (2012). Overexpression of AQP5 in cervical cancer: correlation with clinicopathological features and prognosis. *Med. Oncol.* 29 1998–2004. 10.1007/s12032-011-0095-6 22048942

