# Supplementary figures and images for: Identification of EMT-Related Gene Signatures to Predict the Prognosis of Patients With Endometrial Cancer
Source: Front Genet. 2020 Dec 2;11:582274. doi: 10.3389/fgene.2020.582274 (PMC7738567; doi:10.3389/fgene.2020.582274)

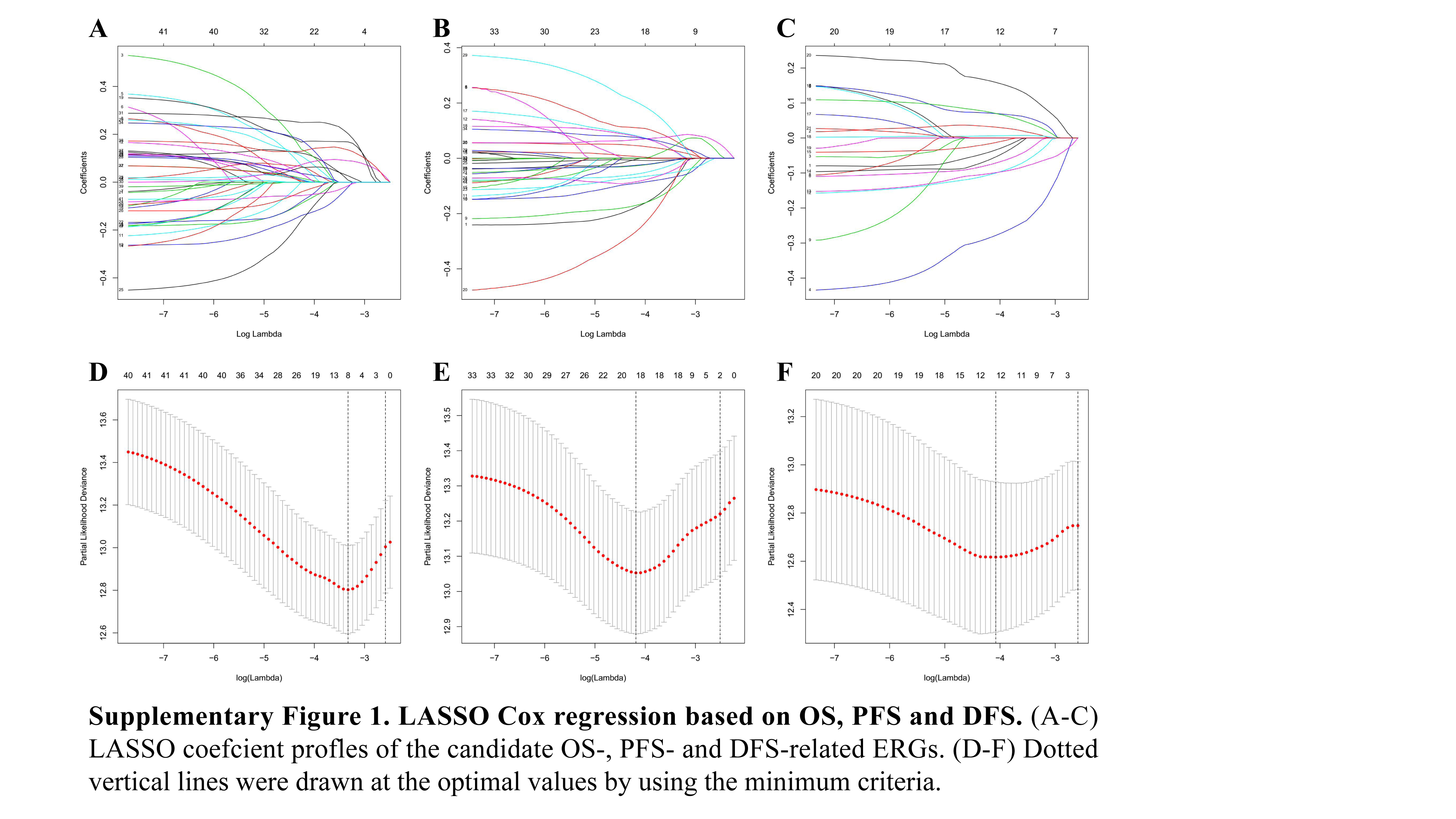

Supplement: Supplementary Figure 1 — LASSO Cox regression based on OS, PFS, and DFS. (A–C) LASSO coefficient profiles of the candidate OS-, PFS-, and DFS-related ERGs. (D–F) Dotted vertical lines were drawn at the optimal values by using the minimum criteria. [file Image_1.TIF]
